# Effect of nitric oxide on renal autoregulation during hypothermia in the rat

**DOI:** 10.1007/s00424-017-1967-1

**Published:** 2017-03-17

**Authors:** Lars Mikael Broman, Mattias Carlström, Örjan Källskog, Mats Wolgast

**Affiliations:** 1 ECMO Centre Karolinska, Department of Pediatric Perioperative Medicine and Intensive Care, Karolinska University Hospital, 171 76 Stockholm, Sweden; 2 Department of Physiology and Pharmacology, Karolinska Institutet, 171 77 Stockholm, Sweden; 3 Department of Medical Cell Biology, Section for Physiology, Uppsala University, 751 23 Uppsala, Sweden

**Keywords:** Autoregulation, GFR, Hypothermia, Nitric oxide, Renal blood flow, Vascular resistance

## Abstract

Hypothermia-induced reduction of metabolic rate is accompanied by depression of both glomerular perfusion and filtration. The present study investigated whether these changes are linked to changes in renal autoregulation and nitric oxide (NO) signalling. During hypothermia, renal blood flow (RBF) and glomerular filtration rate (GFR) were reduced and urine production was increased, and this was linked with reduced plasma cGMP levels and increased renal vascular resistance. Although stimulation of NO production decreased vascular resistance, blood pressure and urine flow, intravenous infusion of the NO precursor L-arginine or the NO donor sodium nitroprusside did not alter RBF or GFR. In contrast, inhibition of NO synthesis by N^w^-nitro-L-arginine led to a further decline in both parameters. Functional renal autoregulation was apparent at both temperatures. Below the autoregulatory range, RBF in both cases increased in proportion to the perfusion ±pressure, although, the slope of the first ascending limb of the pressure-flow relationship was lower during hypothermia. The main difference was rather that the curves obtained during hypothermia levelled off already at a RBF of 3.9 ± 0.3 mL/min then remained stable throughout the autoregulatory pressure range, compared to 7.6 ± 0.3 mL/min during normothermia. This was found to be due to a threefold increase in, primarily, the afferent arteriolar resistance from 2.6 to 7.5 mmHg min mL^−1^. Infusion of sodium nitroprusside did not significantly affect RBF during hypothermia, although a small increase at pressures below the autoregulatory range was observed. In conclusion, cold-induced rise in renal vascular resistance results from afferent arteriolar vasoconstriction by the autoregulatory mechanism, setting RBF and GFR in proportion to the metabolic rate, which cannot be explained by reduced NO production alone.

## Introduction

In our previous studies [5, 6], moderate hypothermia at 28 °C (HT) was found to be accompanied by an approximately 50% reduction of total renal blood flow (RBF), and glomerular filtration rate (GFR) compared with normothermia (NT) at 37.5 °C. Since the blood pressure remained largely unchanged, it was calculated that the renal vascular resistance increased twofold, of which 75% was attributed to active vasoconstriction of, primarily, the afferent arteriole.

In search for the factor(s) underlying this pronounced vasoconstriction, an activation of the sympathetic nervous system, our major cold defence system [17], was an unlikely candidate since direct recordings showed that efferent renal sympathetic nerve activity was decreased [6, 30, 33]. Likewise, surgical renal denervation and α_1_-adrenoceptor blockade were found not to lessen the cold-induced vasoconstriction [6]. A study using micropuncture technique showed that both single nephron GFR, and proximal and distal tubular fluid flow decreased by approximately 50% during HT [5]. The diuresis, however, increased three- to sixfold that could be attributed to a vasopressin-dependent decrease in water reabsorption at the distal tubule and collecting duct [7].

In the present study, we addressed the question of whether the increased arteriolar tone was due (1) to cold-induced inhibition of nitric oxide (NO) production, or (2) to a resetting of the renal autoregulatory mechanism. Concerning the first possibility, it is firmly established that NO plays an important role in the control of vasomotor tone in the renal vascular bed [10, 31]: Inhibition of NO formation by NO synthase inhibitors has been found to increase renal vascular resistance and reduce RBF not only in pressor dosages [2, 25], but also in dosages not affecting the systemic blood pressure [10, 14, 24]. Conversely, infusion of the NO precursor L-arginine and the NO donor sodium nitroprusside (SNP) has been found to lessen renal vasoconstriction [22, 23]. The second alternative considered was that the cold-induced increase in vascular resistance may be due to a metabolism-dependent resetting of the mechanism that determines the blood flow within the autoregulatory range. This might be part of the tubuloglomerular feedback mechanism (TGF) [37], where an increased tubular fluid load, sensed by the *macula densa* cells at the end of the thick ascending limb (TAL) of Henle’s loop leads to constriction of the afferent arteriole, which then decreases glomerular perfusion and filtration, and vice versa*.* In other words, the tubular fluid flow determines RBF. The key point in this consideration would be that the sensitivity of the mechanism may vary from one condition to another [31]; as an example, volume expansion has been found to reduce the TGF sensitivity to allow for a relatively high GFR, whereas dehydration will result in increased sensitivity and hence in a decrease in GFR. Further, it should be noted that according to several previous publications [8, 9, 36, 38], inhibition of NO will increase TGF sensitivity. Therefore, the cold-induced decrease in blood flow and GFR would seem not to be dependent on the TGF.

In the present study, two series of experiments were performed. The aim of the first series was to investigate the hypothesis that the cold-induced vasoconstriction was due to impairment of the formation and/or action of NO. This was examined by investigating the effects of intravenous infusion of the NO precursor L-arginine, and of the NO donor SNP, as well as by inhibition of NO synthesis by N^w^-nitro-L-arginine (L-NNA). In a second series, renal autoregulation was studied during NT and HT both before and after intrarenal infusion of SNP. Finally, in a third series, the regulation of NO homeostasis and oxidative stress was investigated during NT and HT.

## Materials and methods

The studies were performed on 50 male Sprague-Dawley rats (Möllegaards Breeding Center, Copenhagen, Denmark) weighing 340 ± 14 g (mean ± SE). The animals had free access to rodent chow (R36, EWOS, Södertälje, Sweden) and tap water, and housed in rooms at 22 °C with a 12/12 day-night cycle until the experiments began. All animal procedures in this study were in strict adherence to the Guide for the Care and Use of Laboratory Animals as adopted by the U.S. National Institutes of Health and were approved by the Regional Animal Care and Use Committee of Uppsala or Stockholm in Sweden.

The rats were anaesthetised with Inactin® (thiobutabarbital, Byk-Gulden, Konstanz, Germany), administered intraperitoneally in a dose of 120 mg per kg. After tracheostomy, the rat was placed on a servo-controlled heating pad which kept the body temperature stable at 37.5 °C under control conditions and at 28 °C during HT.

Catheters were inserted into the right femoral artery and vein; the arterial catheter to be used for measurements of blood pressure and withdrawal of blood samples, and the venous catheter for infusion of isotonic saline at a rate of 5 mL kg^−1^ h^−1^ to cover fluid losses.

The left kidney was exposed through a flank incision, suspended in a Lucite cup and covered with heated mineral oil to prevent drying. The urine from the left kidney was sampled via a catheter inserted into the proximal ureter. In series II, urine was also collected from the right kidney via a supra-pubic catheter inserted into the bladder. In this series, the autoregulation of the RBF was investigated, hence renal perfusion pressure was set by placing an adjustable clamp around the abdominal aorta proximal to the left renal artery. In series III, blood and urine samples were collected, as described above, during the NT and HT periods, and kidneys were excised at the end of the protocol. All tissue samples were instantly frozen using dry ice and stored (−80 °C) for later analyses (see below).

### Analysis and calculations

GFR was determined as the clearance of ^3^H–inulin (NEN, Boston, MA, USA). For this purpose, 2.5 mCi of this tracer was added to each millilitre of the saline that was infused to cover fluid losses. The radioactivity of ^3^H–inulin in collected plasma and urine samples were measured by liquid scintillation technique.

In series I, the RBF was analysed by Ficks principle by dividing the urinary excretion of inulin with its arteriovenous concentration difference. In order to sample renal venous blood, the renal vein was catheterised with a silicon tube with an outer diameter of 1 mm. This flexible tubing was introduced via a hole in the vein first made by a steel cannula. Ten minutes after puncture, the cannula was withdrawn without any blood loss and replaced with the silicon catheter [1].

In series II, RBF was measured continuously with an ultrasonic flow probe (1RB/T206, Transonic Systems Inc., Ithaca, NY, USA) that was placed around the left renal vein. From these data, the renal vascular resistance under the various conditions was calculated: Experimental data were utilised to calculate vascular conductance (C) since the error in the determination of RBF is generally larger than that of pressures. An error in the very low blood flows recorded at lesser pressures will result in a very large error in the renal vascular resistance (RVR), ΔP/ΔRBF. In the determination of C, on the other hand, the percentage error in C will be the same as that in blood flow. C was calculated by dividing the RBF by the renal perfusion pressure. The latter was obtained as mean arterial pressure (MAP) minus *critical closing pressure*. RVR was then assessed as the reciprocal of this conductance (RVR = 1/C). Continued discussion will be based on resistance in that it is easy to comprehend and because resistances can be added in order to obtain the total resistance.

### Critical closing pressure

**Fig. 1:**
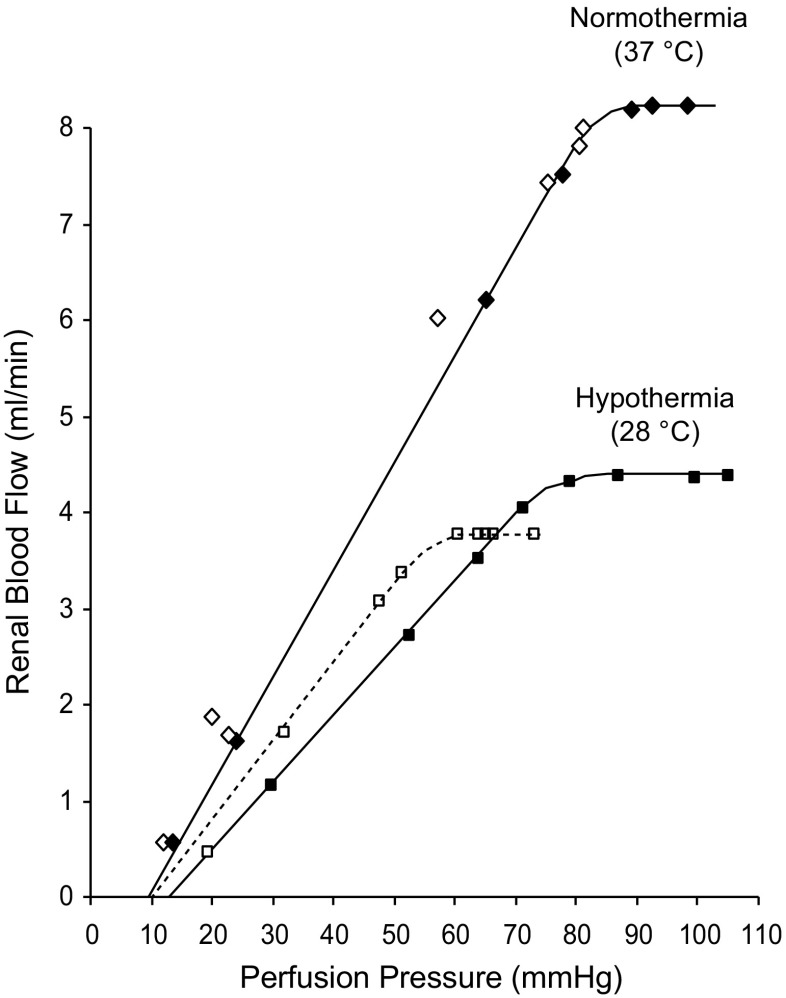
*Autoregulation:* Relation between mean arterial blood pressure (i.e. perfusion pressure) and RBF in one experiment during NT (*upper curves*, *diamonds*), and HT at 28 °C (*lower curves*, *squares*) before (*filled diamonds*/*squares*) and during intrarenal infusion of sodium nitroprusside (*open diamonds*/*squares*)

**Fig. 2:**
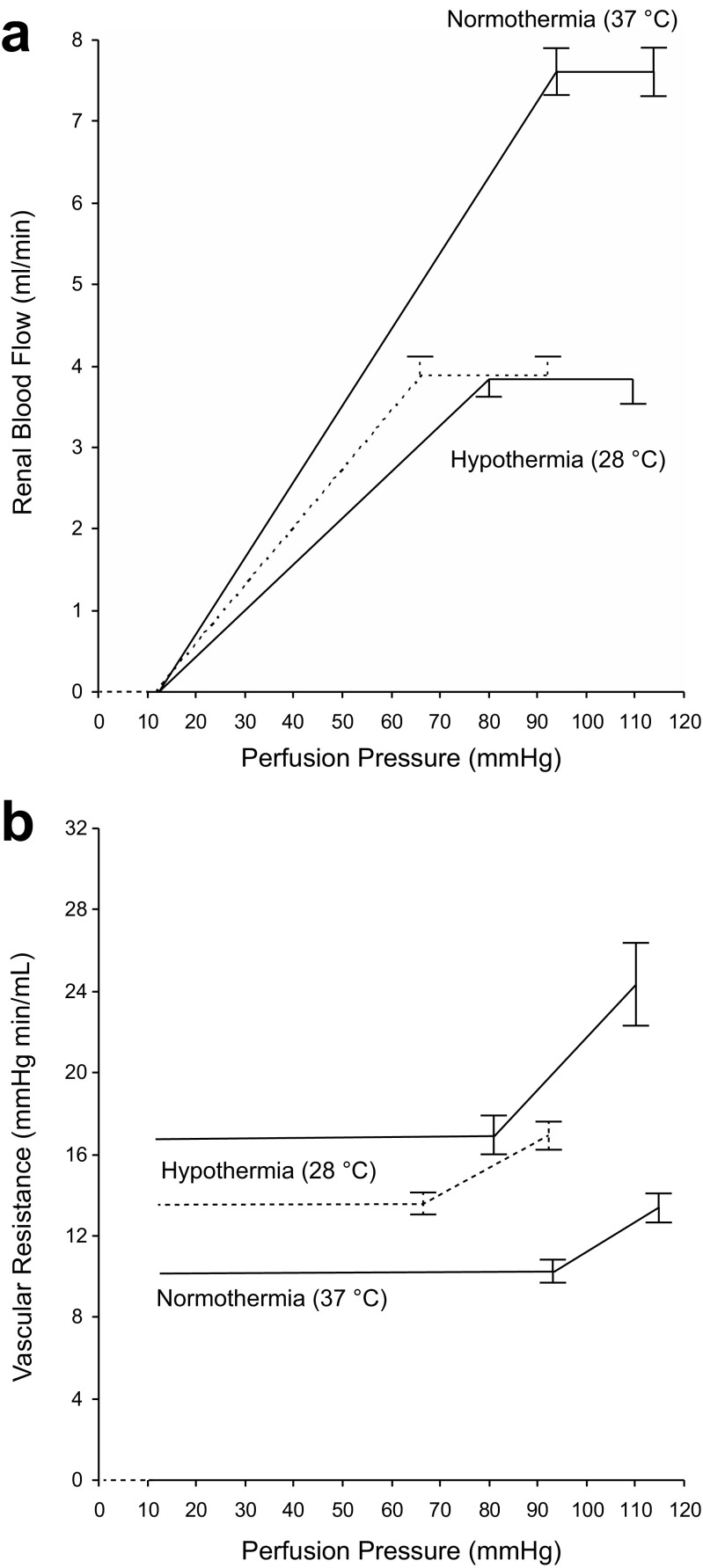
*Autoregulation:* Relation between mean arterial blood pressure (i.e. perfusion pressure) and RBF (**a**) and renal vascular resistance (**b**) in eight experiments during NT, and HT at 28 °C (*lower curves* in **a**; *upper curves* in **b**). During HT the autoregulatory curve is shown both before (*solid line*) and during intrarenal infusion of sodium nitroprusside (*dashed line*). Values are shown as mean ± SE

A certain perfusion pressure is needed to open up and commence blood flow through an organ, e.g. the renal vasculature. The renal interstitial pressure may not be, as for other organs, an equivalent to the intraabdominal pressure (IAP) [21] below which organ perfusion ceases to occur. The abdominal perfusion pressure is considered the difference between the mean arterial blood pressure (MAP) and IAP. Concerning the kidneys, however, it has previously been shown that the pressure in the small intrarenal veins is about 10 mmHg higher than in the extrarenal veins [20]. Thus, best estimate for the closing pressure was used, i.e. the point of intersection by the ascending limb of the autoregulatory curve of the *x*-axis. These curves crossed at 10 mmHg (Figs. 1 and 2a).

### Experimental protocol

#### Series I: intravenous infusion of L-arginine, SNP and L-NNA

The experiments began about 1 h after completion of surgery, when the rat was considered to be in steady state. All the experiments comprised three 20-min periods.

*Period 1* was the control period during which the rat body temperature was set at its normal value of 37.5 °C. After the first period*,* cooling was commenced by turning the servo-controlled heating pad to 28 °C and covering the animal with medical rubber gloves containing crushed ice. When the body temperature had reached 28 °C, the ice was removed and the temperature was maintained by the heating pad. The time taken to reach 28 °C ranged from 60 to 100 min.

*Period 2* was the hypothermic control period that started about 20 min after the body temperature had become stable.

*Period 3* was the hypothermic experimental period, during which either L-arginine (Sigma Labkemi, Stockholm, Sweden), SNP (Nipride, Roche, Basel Schweiz) or L-NNA (N^w^-nitro-L-arginine, Sigma Labkemi) was administered intravenously. L-arginine was given at a rate of 17 mg kg^−1^ min^−1^ preceded by a bolus injection of 100 mg kg^−1^. SNP was given in a dose of 2–7 mg kg^−1^ min^−1^. The dose was set such that MAP did not fall below 95 mmHg. L-NNA was infused in 10 min in a single dose of 20 mg/kg.

A control group of experiments was performed with the same protocol as above but at NT during all three periods.

#### Series II: autoregulation of RBF before and during infusion of SNP

The effect of NO on renal autoregulation was studied by infusing SNP at a dose of 1–4 mg/kg per minute into the left renal artery through a 200-μm polyethylene catheter inserted via a lumbar artery and then advanced with the tip located just inside the renal artery, a technique that enables a very good mixing of the infused substances. In order to prevent coagulation in the catheter before the administration of SNP, saline was continuously infused at a rate of 3 mL/min.

#### Series III: NO homeostasis and oxidative stress

After completing the surgery, a recovery period of 25 min was allowed before starting the experimental protocols. Urine and blood samples were collected after a period with NT (30 min), which was followed by induction of HT as described above. Urine and blood samples were then collected again after a period with HT (30 min), and the kidneys were removed and frozen. A time-matched control group was used where the first NT period was followed by a second NT period. Obtained blood samples were immediately centrifuged at +4 °C (5 min; 4700 *g*) and the plasma was stored at −80 °C until analyses.

##### Cyclic guanosine monophosphate (cGMP)

Plasma content of cGMP was analysed using Direct Biotrak EIA kit (RPN226, GE Healthcare Life Sciences, Uppsala, Sweden), according to manufacturer’s instructions including the acetylation protocol. An inhibitor of cAMP/cGMP phosphodiesterases (IBMX) was added to plasma samples (final conc. 10 μM) before freezing and later processing to prevent degradation of cGMP [18].

##### Nitrate and nitrite

Urine samples were diluted (1:20) in Carrier solution containing 10% methanol before being analysed. Plasma samples were extracted using high-performance liquid chromatography (HPLC)-grade methanol (Chromasolv, Sigma-Aldrich), and the frozen kidney samples were cut and homogenised as described previously [8]. A HPLC system dedicated to assessment of nitrite and nitrate (ENO-20; EiCom, Japan) and attached to an auto-sampler (840, EiCom, Kyoto, Japan) was used. Detailed method description was recently published [28].

##### NADPH oxidase activity

Chemiluminescence technique was used to determine NADPH stimulated superoxide formation in the kidney [18]. In brief, frozen kidney samples were homogenised in PBS with zirconium oxide beads (0.5 mm) in the bullet blender. The supernatant was collected after centrifugation at 4 °C for 20 min at 2000 *g*. Supernatant containing 500 μg of protein was added into a test tube with 1 ml PBS and 5 μmol/L lucigenin and the reaction was started by the addition of 100 μmol/L NADPH. The chemiluminescence signal was calculated over a period of 3 min and corrected by tissue amount.

##### Statistics

All data are given as mean ± SE, and a *p* value less than 0.05 was considered to be a statistically significant difference. Two-way analysis of variance (ANOVA) with repeated measures was performed, and for comparison between groups in the same period, Tukey’s post-test was applied. For single comparison of data from the left and right kidney, paired *t* test was used. For the analyses of NO homeostasis (plasma, kidney, urine) and NADPH oxidase activity (kidney), two-way ANOVA with repeated measures and Sidak’s test (multiple comparisons) or non-parametric Kolmogorov-Smirnov test (single comparison) were used.

## Results

### Series I: intravenous infusion of L-arginine, SNP and L-NNA

During hypothermia at 28 °C, see Table 1, RBF decreased by more than 50%. Since MAP showed little change, the reduction in RBF was brought about by a more than twofold increase in renal vascular resistance (RVR). In the second hypothermic period (period 3), the vascular resistance remained essentially unchanged after intravenous infusion of either the NO precursor L-arginine or the NO donor SNP. Inhibition of NO synthesis by L-NNA led to a further, marked increase in RVR. Consequently, RBF decreased from 5.2 ± 1.25 to 1.4 ± 0.38 mL/min (*p* < 0.001) and GFR from 0.8 ± 0.2 to 0.2 ± 0.05 mL/min (*p* < 0.001). These changes were obtained despite of the fact that L-NNA increased MAP by about 20%. The same pattern was observed in normothermia (Table 2), where L-arginine and SNP only evoked modest changes in RVR. After inhibition of NO synthesis by L-NNA, however, the resistance increased with a concomitant reduction in both RBF and GFR although MAP increased by 30%.

**Table 1:** The effects of L-arginine, SNP and L-NNA given intravenously before and during period 3, on renal function during HT

Parameter	Period 1 NT	Period 2 HT	Period 3, HT effects of vasoactive substances	
MAP (mmHg)	124 ± 3.3	120 ± 4.9	after L-ARG	116 ± 5.6
	122 ± 2.3	121 ± 3.4	after SNP	98 ± 0.7**^ooo^
	113 ± 2.9	121 ± 4.4	after L-NNA	148 ± 8.9***^ooo^
GFR (mL/min)	1.43 ± 0.09	0.54 ± 0.04***	after L-ARG	0.63 ± 0.05***
	1.49 ± 0.05	0.71 ± 0.06***	after SNP	0.61 ± 0.04***
	1.40 ± 0.24	0.82 ± 0.22**	after L-NNA	0.22 ± 0.05***^ooo^
RBF (mL/min)	10.5 ± 1.5	3.7 ± 0.2***	after L-ARG	4.4 ± 0.5***
	8.9 ± 0.7	4.0 ± 0.6***	after SNP	3.1 ± 0.3***
	10.2 ± 1.2	5.2 ± 1.2***	after L-NNA	1.4 ± 0.4***^ooo^
Urine flow (μL/min)	2.3 ± 0.6	5.3 ± 1.2	after L-ARG	7.3 ± 3.0**
	2.1 ± 0.4	3.5 ± 0.9	after SNP	1.5 ± 0.4
	1.7 ± 0.3	6.7 ± 3.0**	after L-NNA	2.0 ± 0.6°
*U*_Na_V (μmol/min)	0.13 ± 0.06	0.36 ± 0.18	after L-ARG	0.64 ± 0.37*
	0.09 ± 0.02	0.21 ± 0.07	after SNP	0.09 ± 0.05
	0.08 ± 0.01	0.52 ± 0.24**	after L-NNA	0.19 ± 0.10^o^
Vascular resistance (mmHg min/mL)	10.9 ± 1.7	29.7 ± 2.0**	after L-ARG	24.1 ± 1.6
	12.6 ± 0.9	27.8 ± 5.1***	after SNP	28.4 ± 2.6
	10.1 ± 1.2	21.3 ± 5.0**	after L-NNA	98.5 ± 11.0**^oo^

Values are shown as mean ± SE. *n* = 5 in the L-ARG group, *n* = 6 in the SNP group and *n* = 6 in the L-NNA group. The closing pressure (10 mmHg) has been subtracted when calculating vascular resistance

*MAP* mean arterial blood pressure, *GFR* glomerular filtration rate, *L-ARG* L-arginine, *SNP* sodium nitroprusside, *RBF* renal blood flow, *U*_*Na*_ *V* renal sodium excretion

*p < 0.05, **p < 0.01, ***p < 0.001, versus period 1; °*p* < 0.05, ^oo^ *p* < 0.01, ^ooo^ *p* < 0.001 versus period 2

**Table 2:** The effects of L-arginine, SNP and L-NNA given intravenously before and during period 3, on renal function under control conditions at 37.5 °C

Parameter	Period 1 NT	Period 2 NT	Period 3, NT effects of vasoactive substances	
MAP (mmHg)	116 ± 3.3	116 ± 2.9	after L-ARG	111 ± 3.4
	121 ± 3.1	116 ± 2.5**	after SNP	97 ± 0.6**^ooo^
	114 ± 4.0	113 ± 3.4	after L-NNA	148 ± 4.1***^ooo^
GFR (mL/min)	1.40 ± 0.10	1.52 ± 0.11	after L-ARG	1.42 ± 0.10
	1.31 ± 0.13	1.48 ± 0.08	after SNP	1.41 ± 0.06
	1.53 ± 0.08	1.54 ± 0.06	after L-NNA	1.11 ± 0.11***^ooo^
RBF (mL/min)	9.1 ± 1.0	9.5 ± 0.7	after L-ARG	10.0 ± 0.9
	9.4 ± 0.8	9.7 ± 1.4	after SNP	9.6 ± 1.1
	10.8 ± 1.2	11.5 ± 2.0	after L-NNA	5.3 ± 0.5***^ooo^
Urine flow (μL/min)	1.3 ± 0.2	2.1 ± 0.4*	after L-ARG	3.5 ± 0.7*
	2.0 ± 0.2	3.0 ± 0.2**	after SNP	2.7 ± 0.7
	2.2 ± 0.2	2.6 ± 0.3	after L-NNA	7.0 ± 1.9***°°
*U*_Na_V (μmol/min)	0.06 ± 0.01	0.13 ± 0.02**	after L-ARG	0.28 ± 0.07*
	0.12 ± 0.03	0.20 ± 0.05**	after SNP	0.23 ± 0.07
	0.15 ± 0.03	0.18 ± 0.04	after L-NNA	0.27 ± 0.13
Vascular resistance (mmHg min/mL)	11.6 ± 0.8	11.2 ± 1.0	after L-ARG	10.1 ± 1.1
	11.7 ± 1.2	10.9 ± 2.4	after SNP	9.2 ± 1.3
	9.6 ± 0.6	9.0 ± 1.4	after L-NNA	26.0 ± 2.3***^ooo^

Values are shown as mean ± SE. *n* = 6 in the L-ARG group, *n* = 4 in the SNP group and *n* = 5 in the L-NNA group. The closing pressure (10 mmHg) has been subtracted when calculating the vascular resistance

*MAP* mean arterial blood pressure, *GFR* glomerular filtration rate, *L-ARG* L-arginine, *SNP* sodium nitroprusside, *RBF* renal blood flow, *U*_*Na*_ *V* renal sodium excretion

*p < 0.05, **p < 0.01, ***p < 0.001 versus period 1; °°*p* < 0.01, ^ooo^ *p* < 0.001 versus period 2

### Series II: autoregulation of RBF before and during infusion of SNP

Figure 1 depicts the relation between MAP and RBF blood flow from an experiment in one specimen during NT and HT at 28 °C, before and during intrarenal infusion of SNP. Figure 2 summarises data from all autoregulation curves obtained, and effects of HT on renal function before and after intrarenal infusion of SNP are summarised in Table 3. Autoregulation curves in Fig. 2a were constructed so that the values falling on the ascending portion of the curves were fitted to one line and those within the autoregulatory range to a second, horizontal line. The lower end of the autoregulatory range was defined as the point of intersection between these two lines. The kidney was able to autoregulate the blood flow under normal temperature and hypothermic conditions. The main difference seen was that RBF decreased from 7.6 ± 0.3 mL/min under NT to 3.9 ± 0.3 mL/min during HT. In Fig. 2b, the perfusion pressure was set against RVR. During HT, the vascular resistance at perfusion pressures below the lower point of inflexion was substantially elevated as compared to NT. This increase in resistance was partially released when SNP was infused via the renal artery (dashed line). At blood pressures in the range where autoregulatory activity occurred during HT, the slope was less steep during SNP infusion than under conditions without the NO donor. During NT, no difference in slope was expected since both autoregulatory curves (w/wo SNP) overlapped at NT (see Fig. 1).

**Table 3:** The effect of HT on renal function during control conditions and at 28 °C, before and during intrarenal infusion of SNP

Parameter	Period 1 NT	Period 2 HT	Period 3, HT intrarenal infusion of SNP
MAP (mmHg)	116 ± 3.3	114 ± 2.7	89 ± 1.6***°°°
GFR (mL/min)	L 1.28 ± 0.15	0.76 ± 0.18**	0.58 ± 0.06***
	R 1.40 ± 0.16	0.62 ± 0.08***	0.58 ± 0.06***
RBF (ml/min)	L 7.6 ± 0.3	3.9 ± 0.3***	4.0 ± 0.3***
Urine flow (μL/min)	L 2.0 ± 0.3	5.2 ± 0.8**	6.9 ± 0.9***°
	R 1.6 ± 0.3	4.7 ± 0.6**	4.0 ± 0.9*
U_Na_V (μmol/min)	L 0.09 ± 0.02	0.13 ± 0.05	0.45 ± 0.14*°°°

Values are shown as mean ± SE. *n* = 8/group

*SNP* = sodium nitroprusside, *MAP* mean arterial blood pressure, *GFR* glomerular filtration rate, *L* left kidney, *R* right kidney, *RBF* renal blood flow, *U*_*Na*_ *V* renal sodium excretion

*p < 0.05, **p < 0.01, ***p < 0.001 versus period 1; °*p* < 0.05, ^ooo^ *p* < 0.001 versus period 2

**Fig. 3:**
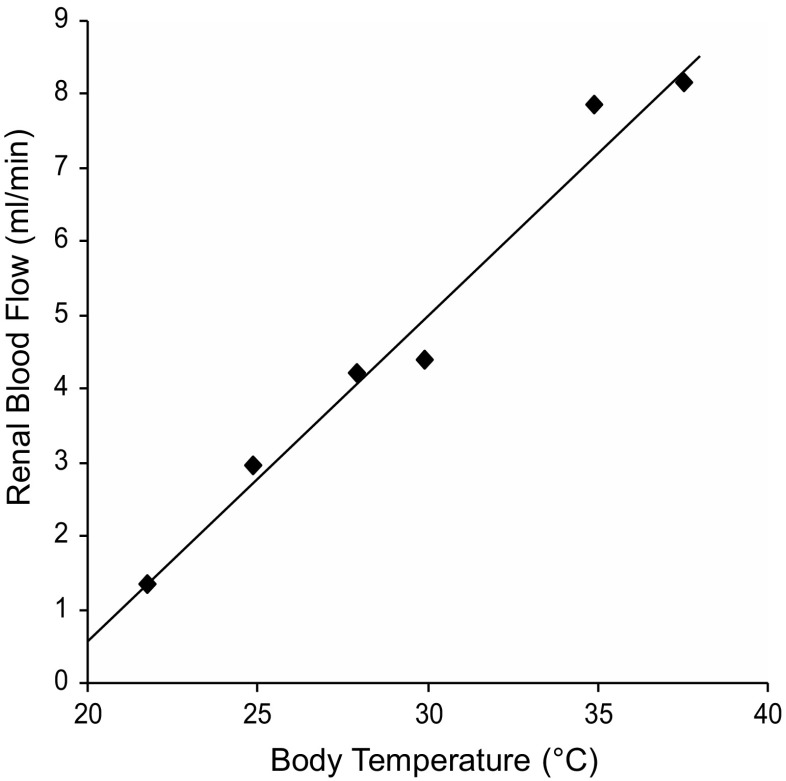
Representative presentation of the relation between body temperature and RBF in one of the experiments during HT.

As illustrated in Fig. 3, RBF decreases in proportion to the body temperature. Since MAP largely remained unchanged between periods 1 and 2, the reduction in RBF was due to a rise in RVR.

At the first ascending portion, RBF was found to increase in proportion to arterial pressure, where each rise in perfusion pressure, ΔP, was followed by a rise in blood flow, ΔRBF. The slope of the curve, ΔP/ΔRBF, is in these cases an indirect estimate of the vascular resistance at any point at the ascending limb as supported by the findings depicted in Fig. 1. It should be noted that all the four curves crossed the *x*-axis at a “critical closing pressure” of about 10 mmHg. This was also taken into account for in the sense that the renal perfusion pressure was calculated by subtracting 10 mmHg from MAP. In practice, the vascular resistances were calculated by dividing the renal perfusion pressure by the blood flow at the lower limit of the autoregulatory range.

*During normothermia*, the “passive” vascular resistance calculated from the lower end of the autoregulatory range (or from the slope of the ascending portion) was 10.8 ± 0.58 mmHg min mL^−1^. At normal blood pressure, the corresponding resistance was13.4 ± 0.89 mmHg min mL^−1^. The increase in resistance brought about by the autoregulatory vasoconstriction was thus 13.4–10.8 = 2.6 mmHg min mL^−1^ (*p* < 0.01).

During infusion of SNP (Fig. 1), neither the slope of the ascending part of the curve, nor the critical closing pressure changed. This indicated that the vascular bed was “passive”. Whether or not SNP affected the blood flow *within* the autoregulatory range is not known, as MAP decreased to pressures below the autoregulatory limit.

*During hypothermia*, the “passive” vascular resistance estimated from the lower end of the autoregulatory range (or from the slope) was estimated to 16.5 ± 1.35 mmHg min mL^−1^. At normal MAP, RVR increased to 24.0 ± 2.29 mmHg min mL^−1^. The rise in resistance offered by the autoregulatory mechanism was thus 24.0–16.5 = 7.5 mmHg min mL^−1^, a figure three times higher than during normal conditions.

**Fig. 4:**
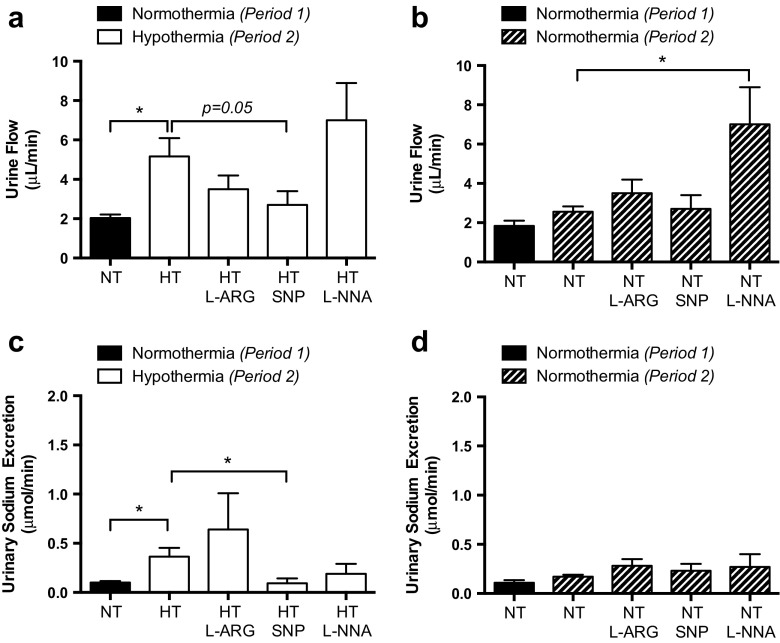
Summarised data on urine flow (**a**, **b**) and sodium excretion (**c**, **d**) during NT (period 1) followed by a second period with either NT or HT (28 °C). During period 2, the effects of intrarenal infusion of L-arginine, sodium nitroprusside, and L-NNA are shown. *p < 0.05. Values are shown as mean ± SE. *n* = 8/group

After SNP, the resistance obtained from the lower end of the autoregulatory range decreased from 16.5 to 13.4 mmHg min mL^−1^ (*p* < 0.01). The latter figure was considered to reflect the true “passive” resistance of the renal vascular bed during HT (Figs. 1 and 2). The blood flow was not altered within the autoregulatory range by donation of NO. The approximate twofold increase in resistance, or actually 50% decrease in oxygen delivery during HT at 28 °C, indicates that RBF is determined by the renal metabolic rate, which is half the normal at these temperatures [4]. In one experiment, RBF data was obtained during the cooling phase, suggesting such linear relationship (Fig. 3). Data on urine flow and sodium excretion obtained during NT and HT, before and after infusion of vasoactive compounds, are shown in Fig. 4a–d. In brief, HT was associated with increased urine production and sodium output compared with NT, which were restored by SNP infusion. Inhibition of NO synthase with L-NNA had no significant effect during HT, whereas NO inhibition significantly increased urine flow during NT. Although there were no profound effects of HT on blood pressure, averaged MAP was significantly (*p* = 0.005) higher during period 2 with HT (121.0 ± 0.3 mmHg; *n* = 17) compared with the same period with NT (115.0 ± 1.0; *n* = 15). Stimulation of NO production with SNP reduced blood pressure and inhibition of NO synthesis with L-NNA increased blood pressure in both groups, abolishing the differences in MAP between NT and HT. Taken together, this suggests that HT is associated with abnormal NO bioavailability.

### Series III: NO homeostasis and oxidative stress

**Fig. 5:**
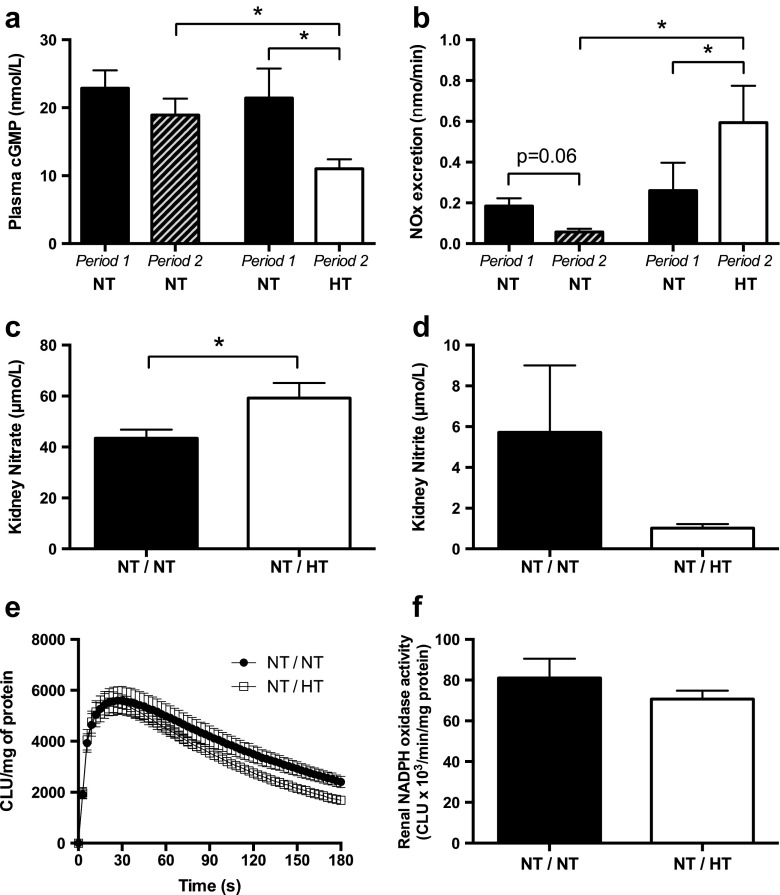
Effects of hypothermia on NO homeostasis and oxidative stress. Plasma cGMP (**a**) and nitrate + nitrite (NOx) excretion (**b**) in rats during two consecutive periods with normothermia (NT) or in rats where first period of NT was followed by hypothermia (HT). Renal concentrations of nitrate (**c**) and nitrite (**d**) in rats exposed to NT followed by NT or NT followed by HT. Traces (**e**) and grouped data (**f**) of renal NADPH oxidase-derived superoxide production in rats exposed to NT followed by NT or NT followed by HT. *p < 0.05. Values are shown as mean ± SE. *n* = 5/group

Plasma levels of cGMP were reduced *(*Fig. 5a), and the urinary excretion rate of nitrate and nitrite anions (NOx) was increased during HT (Fig. 5b), indicating reduced NO signalling. However, systemic NOx levels were not different between the groups during the different periods (NT period 1, 22.26 ± 4.6; NT period 2 31.51 ± 5.4; NT period 1, 28.18 ± 5.3; HT period 2 27.64 ± 2.9; *n* = 5/group). To further investigate the regulation of NO markers in the kidney, we analysed nitrate and nitrite levels in the kidney. Increased metabolism of NO would be associated with accumulation of the stable nitrate anion whereas levels of the more short-lived nitrite anion should decrease. Indeed, the renal nitrate concentration (Fig. 5c) was significantly increased whereas nitrite content (Fig. 5d) tended to be decreased after HT compared with NT. Although not statistically significant, the nitrate-to-nitrite ratio was also increased in HT kidneys compared with NT kidneys (68 ± 6 vs. 39 ± 12, *p* = 0.08, *n* = 5/group). Reduced NO bioavailability in the kidney could be linked to decreased production or increased metabolism via its interaction with reactive oxygen species. NADPH oxidase is a major source of superoxide generation in the kidney; however, its activity was not changed by HT (Fig. 5e–f).

## Discussion

In agreement with our previous studies [5, 6] and findings from other laboratories [4, 11, 16, 33, 39], HT was found to be accompanied by a decline in RBF to about half of that under normal conditions. Since MAP remained largely unchanged, the depression of the blood flow resulted from a twofold increase in renal vascular resistance.

In the search for the factor(s) underlying the cold-induced vasoconstriction, the first series of experiments in the present study were designed to investigate whether the increased tone was dependent on a cold-induced inhibition of NO, which in recent years has been proved to be one of the main determinants of vascular tone [15, 27, 29, 32]. Stimulation of the synthesis of NO by administration of its precursor L-arginine or a NO donor as SNP has generally been found to increase RBF, although not GFR [22, 23, 29]. Conversely, blocking NO by NO synthase inhibitors has been found to decrease RBF and GFR despite of the fact that MAP increases.

In the present study, intravenous infusion of the NO precursor L-arginine during both normo- and HT did not significantly affect RBF or GFR. Administration of the NO donor SNP was more effective in the sense that it reduced MAP during both these conditions. During HT, SNP was also able to decrease vascular resistance, however. The failure of L-arginine to affect renal vessels was to be expected, i.e. since it seems unlikely that cold should deplete the stores of this precursor, and the effect of reduced metabolism would seem more likely. However, higher viscosity due to haemo-concentration and physical properties may promote increased shear stress at the endothelial wall and may be expected to enhance the formation of NO [15, 29]. Still, NO would seem important since inhibition of NO synthesis by L-NNA increased RVR five-fold in addition to the twofold increase caused by the HT itself.

From the present second series on the pressure-blood flow relationship, it was evident that the autoregulatory mechanism remained functional during HT. This has previously only been described in the dog [11]. It seems that the setting of the autoregulatory mechanism is temperature dependent such as to adjust the blood flow according to the metabolic rate. The “setting” mechanism seems powerful since intrarenal infusion of an effective NO donor like SNP was unable to affect RBF at pressures within the autoregulatory range, only below this range.

At the first ascending portion of the pressure-flow relationship, the blood flow was linearly related to perfusion pressure up to the lower limit of the autoregulatory range. The fact that vascular resistance remained unchanged across the whole of the ascending portion of the pressure-flow relationship suggests a “passive” vasculature with unchanged vessel diameter(s). At very low perfusion pressure, autoregulation is discontinued, most likely since the vessels are fully dilated. However, it will not exclude changes in the vasculature in question. Within the autoregulatory range, the blood flow was found to remain remarkably constant with a standard error of as little as 0.3 mL/min, a constancy obviously due to the autoregulatory vasoconstriction. We may thus express the autoregulatory capacity as the rise in resistance when effective perfusion pressure increases from the lower end of the autoregulatory range up to normal pressure.

All the autoregulation curves crossed the *x*-axis at 10 mmHg (Figs. 1 and 2a), thus regarded as the closing pressure. In the interpretation of this phenomenon, it should be emphasised that the curves were perfectly linear with no sign of splay in the region close to the crossing with the *x*-axis. As previously shown under NT [20], the pressure in the small intrarenal veins is constantly about 10 mmHg higher than in extrarenal veins probably due to collapse of the distal part of the intrarenal veins creating an “outflow resistance”. For very low perfusion pressures up to 10 mmHg, all of the intrarenal veins may be expected to be collapsed. In agreement with this view, a rise in the central venous pressure above 10 mmHg eliminates the pressure drop. The physiological significance of the distinct pressure drop is not known, but it may act to stabilise the peritubular capillary pressure at 10–13 mmHg, and hence one of the Starling forces which governs peritubular capillary fluid uptake. Therefore, it is reasonable to subtract the critical closing pressure of 10 mmHg from the MAP. Using this definition, each rise in perfusion pressure, ΔP, anywhere at the ascending portion of the curves is followed by a correlating increase in RBF, ΔRBF. This feature is typical for a “passive” vasculature with unchanged diameters; it does not, however, exclude a change in the size of the vessels. The slope of the ascending portion will equal ΔP/ΔRBF, which thus is a measure of the vascular resistance. Within the autoregulatory range (plateau), on the other hand, the blood flow was found to remain virtually constant.

Under normothermic conditions, the total vascular resistance almost doubled along the plateau. Regarding the first ascending portion of the pressure-flow relationships, considered to reflect a “passive” behaviour of the vasculature, the resistance was estimated to 10.8 mmHg min mL^−1^ at NT. After cooling, RVR increased to 16.5 mmHg min mL^−1^. HT thus increased “passive” resistance by 5.7 mmHg min mL^−1^.

Regarding the autoregulatory response, the rise in resistance when passing from the lower end of this range to normal pressures was found to be 2.6 mmHg min mL^−1^ during NT increasing to 7.5 mmHg min mL^−1^ during HT (i.e. difference of 4.9 mmHg min mL^−1^). HT thus enhanced the autoregulatory response leading to vasoconstriction, which may explain the reduction in RBF and GFR during moderate HT. The resetting of TGF seems plausible as supported by earlier data from our department [5] where single nephron GFR measurements show a 50% reduction (approximately) in both proximal and distal tubular fluid flow during HT compared to NT. Fractional fluid reabsorption was in fact unaffected by HT, and the increased diuresis during HT could be attributed the distal tubule and collecting duct as shown in a previous study [7], in which Arg-vasopressin (AVP) secretion was found to decrease with a concomitant increase in water loss, and a bolus of AVP abolished the cold-induced diuresis. The NO pathways may well be involved also in these water handling mechanisms; Ota et al. [30] demonstrated that NO-mediated signalling in the central nervous system increase the secretion of ADH, and vice versa. Although neural NO bioavailability was not assessed in the current investigation, we found that systemic level of cGMP was reduced by almost 50% during HT, clearly suggesting reduced NO signalling. Concerning renal metabolic pathways for NO, a decrease in NO production during HT was supported by increased urinary excretion of the NO metabolites nitrate and nitrite (i.e. NOx). In the kidney, enzymatic reduction of nitrite to NO is associated with preglomerular vasodilatation and reduction of NADPH oxidase-mediated oxidative stress. Here, we show that renal nitrite content decreased whereas nitrate increased (Fig. 5c–d), suggesting increased oxidation of NO. Although some previous studies have suggested HT-mediated modulation of oxidative stress in models of ischemia or hypoxia [34, 35], the observed reduction of NO bioavailability in the current study cannot be explained by increased NADPH oxidase activity (Fig. 5b).

Regarding the site of the cold-induced vasoconstriction, the “passive” resistance will probably affect both pre- and postglomerular vessels, each of them contributing with 2.85 mmHg min mL^−1^. This is partly due to increased viscosity from temperature per se, and a slight physiological increase in haematocrit [3, 5]. The active component of 4.9 mmHg min mL^−1^ will only affect preglomerular resistance, i.e. since it is generally agreed that renal autoregulation is the result of changes in these vessels [20, 29]. Thus, to the total cold-induced rise in renal vascular resistance of 10.6 mmHg min mL^−1^, the preglomerular vessels will contribute with 70% (7.75 mmHg min mL^−1^) and the postglomerular with 30% (2.85 mmHg min mL^−1^).

It should be noted that if the pre- and postglomerular increase in resistance was the same, the glomerular capillary pressure would be expected to remain unaltered. If the largest part occurs at the preglomerular vessel segments, however, the glomerular capillary pressure would decrease. The latter is also in accordance with the data of Broman and Källskog 1995 [5] reporting a fall in the glomerular capillary pressure from 56.7 ± 0.6 mmHg during NT to 46.5 ± 1.3 mmHg during HT. The authors also calculated the contributions of the pre- and postglomerular resistances showing that 70% of the HT induced increase in resistance was due to preglomerular vessels, i.e. the same fractions obtained from the present data.

During NT, intra-arterial infusion of SNP did not affect the ascending portion of the curves. The data produced the same line as during normal conditions. Since SNP also decreased MAP, the effect of SNP within the autoregulatory range is not known.

During HT (Fig. 2b), during SNP infusion, the “passive” resistance at the ascending portion of the curves showed an increase to 13.3 mmHg min mL^−1^. This is in fact the same as during NT considering that the blood viscosity at 28 °C is 25% higher than at 37.5 °C [3, 12]. Evidently, SNP was able to unmask the true “passive” resistance during HT, the difference between the data before and after SNP was 3.1 mmHg min mL^−1^. SNP had no effect on the autoregulatory vasoconstriction. As seen in Fig. 2b, still during HT, the slope of the active part of the autoregulation curve is more steep before SNP infusion then after, indicating less active vasculature since the autoregulation in the latter case also is acting accordingly but from a lower perfusion pressure, with the *default* goal—to maintain renal perfusion flow according to metabolic demand. Thus, the present investigation revealed that the autoregulatory vasoconstriction was able to override the vasodilatory action of NO. This may also explain the failure of intravenous infusion of SNP to alter RBF investigated in our first series of studies. In contrast, blockade of the NO synthesis by L-NNA was able to further decrease the blood flow also within the autoregulation range.

The present study specifically investigating the role of the NO pathway does not solve all the questions concerning the underlying mechanism(s) contributing to the resetting of the renal autoregulation during HT. However, our findings add to current understanding in the sense that the setting of this mechanism is dependent on the metabolic rate as shown in Fig. 3. Although a modulatory role should not be excluded, it is clear that the setting is not mediated via NO since administration of SNP or L-arginine did not significantly change the autoregulatory plateau. The latter finding may also be of clinical significance in the sense that administration of, e.g. SNP to lessen the work load of the heart can be expected not to compromise RBF. Furthermore, since the NO donor shifted the autoregulatory plateau to the left by 10–15 mmHg, the RBF will remain unaffected even if MAP would decrease with 10–15 mmHg. It is concluded that the twofold increase in renal vascular tone seen during HT at 28 °C is mainly due to a temperature-dependent resetting of renal autoregulation, which cannot be explained by a reduction of endogenous synthesis of NO alone.

Our group had earlier mapped the resistance pattern along the renal microcirculation during hypothermia [5]. The results showed that the dominating part of the increase in RVR could be addressed to the preglomerular vessels. At hypothermia, renal sympathetic tone was substantially decreased, promoting reduced vascular tone, and increase in sodium and water excretion [6]. It has been proposed by Ito and Abe 1997 [19], and may be argued, that under normal circumstances the mechanisms regulating afferent and efferent vascular tone are different. On the afferent side, the myogenic response and the TGF mechanisms dominate in maintaining GFR, NO being the main modulator. Prostaglandins (PG) would be more important for efferent arteriole, but NO and AngII are also important. Concerning PGs, an increase in renal PG synthesis would promote natriuresis by inhibiting sodium and water reabsorption. It has been shown, at least in mice, that there is a parallel water handling system via PGE2 and the EP4 receptor. A downregulation of aquaporin 2 by less PGE2 receptor EP4 stimulation leads to water dieresis [26]. The vasopressin system, however, works in parallel and was neither down- or upregulated in EP4 knock-out mice compared to wild type; both genetic types increased urine concentration to the same extent after an injection of desmopressin (dDAVP). In the series of studies from our department [5–7], the cold-induced diuresis was found to be due to a decrease in AVP excretion, and a single dose of dDAVP completely reversed the cold-induced diuresis [7]. This strongly suggests PGs not to be contributing to the tubular changes in water handling during moderate hypothermia. Thus, in the light of the current results concerning the NO system, it is less likely that the renal blood flow alterations during hypothermia are PG-dependent.

A limitation of the present study is that a pharmacological approach was used to target NO signalling, and it is difficult to know the effectiveness of the doses used (e.g. sufficient amount reaching the vascular smooth muscle). Although technically more challenging future studies could benefit from using a genetic approach (e.g. targeting eNOS signalling), other potential candidates, apart from NO that would be of interest for future hemodynamic investigation since they are known to modulate renal autoregulation [10], include signalling by prostaniods (prostaglandin, thromboxane and prostacyclin), and possibly altered signalling by endothelium-derived hyperpolarizing factor [13].
